# Supplementary material for: Development, implementation and evaluation of the online Movement, Interaction and Nutrition for Greater Lifestyles in the Elderly (MINGLE) program: The protocol for a pilot trial
Source: PLoS One. 2022 May 12;17(5):e0267581. doi: 10.1371/journal.pone.0267581 (PMC9097998; doi:10.1371/journal.pone.0267581)
Supplement: S2 File — (DOCX) [file pone.0267581.s002.docx]

Study Protocol

The development, implementation and evaluation of an online Movement, Interaction and Nutrition for Greater Lifestyles in the Elderly (MINGLE) program for people with age-related macular degeneration.

Project Team Roles & Responsibilities

**Chief Investigator**

Ms Diana Tang; Research Fellow, Macquarie University

Project Responsibilities: Direct supervision of the research assistant and any students involved in the study; overseeing all project activities; write up and dissemination of study findings.

**Co-Investigators**

Dr Rona Macniven; Research Fellow, Macquarie University

Project Responsibilities: Supporting the Chief Investigator in the supervision of the research assistant and any students involved in the study; overseeing all the physical activity component; write up and dissemination of study findings.

Associate Professor Charlotte Jones; University of British Columbia

Project Responsibilities: Supporting the Chief Investigator in the supervision of the research assistant and any students involved in the study; write up and dissemination of study findings.

Professor Bamini Gopinath; Cochlear Chair in Hearing and Health, Macquarie University

Project Responsibilities: Supporting the Chief Investigator in the supervision of the research assistant and any students involved in the study; assisting in the write up and dissemination of study findings.

Research Sites

Sydney West Retina, a private eye clinic located in Westmead NSW, will be the site for participant recruitment for the MINGLE study.

Resources

**Personnel:** Research Assistant to conduct Phase 1 and 2 of the project, and also assist with data entry, data analysis, and supervision of any research students.

**Consumables:** Postage costs and printed material i.e. consent form, pre-post questionnaire.

**Equipment:** Two audio recording devices to allow the research team members to conduct the semi-structured interviews (Phase 1) and transfer audio recordings to the transcribing service.

**Services:** Verbatim transcribing service

**Funding:** The MINGLE study has secured funding from the Macular Disease Foundation Australia to the value of $45,466 for 1 year.

Background

Age-related macular degeneration (AMD) is the leading cause of vision loss and blindness in developed countries [1]. Lifestyle risk factors (poor diet and physical inactivity) are associated with an increased risk of AMD development and progression [2–4]. AMD-related vision loss is also associated with an increased risk of falls and poorer mental health status [5,6]. Concerningly, depression has even been shown to affect individuals experiencing minimal vision loss [5]. One such study, of mostly AMD patients, reported that 22% of participants were depressed and 23% suffered from anxiety [5]. Rates of depression are also influenced by feelings of loneliness [7], where the prevalence of loneliness in older adults with a vision impairment is significantly greater than older adults with normal vision [6]. Persistent loneliness can also lead to poorer lifestyle behaviours [8]and poorer health outcomes [8–11]. Therefore, evidence-based intervention programs to minimise these risk factors are critical to reducing overall disease burden.

Lifestyle interventions that include education, behaviour change and/or group support components can be effective in reducing depression and loneliness in older adults [12,13]. One example is the ‘Walk N’ Talk for your Life (WTL) program that was developed in 2014 by CI A/Prof Jones for low-income older adults in Canada [14]. The 12-week WTL program includes group walking; resistance training and balance exercises; and discussion of health topics [14]. Since its inception, >300 older adults have participated in this community-based, student and volunteer-run program across multiple locations [14]. With the success of WTL, a virtual adaptation of WTL hosted over Zoom (WTL-Z) was developed to address the safety concerns related to COVID-19, particularly amongst vulnerable populations such as older adults [15]. Unpublished pilot data involving 35 older adults in the United Kingdom has shown that WTL-Z significantly reduced participant loneliness by 20% (p=0.048) and significantly improved depressive symptoms by 35% (p=0.001) [15]. Written participant feedback also indicated that WTL-Z was feasible and well-received by the participants. Amongst people with AMD, positive participant responses to virtual programs has also been reported in a recent evidence-based telehealth nutrition intervention to improve the dietary intakes of people with AMD [16]. This telehealth intervention involving the provision of an evidence-based participant workbook on nutrition and AMD links and monthly individual phone calls with a dietitian over four months, significantly improved the participants’ dietary intake of nutrient-rich dark green leafy vegetables and legumes and reduced consumption of packaged and processed foods. [16]

Therefore, to provide more holistic support to address the risks of loneliness, depression, falls and physical inactivity associated with AMD as well as improve the dietary intakes of key food groups for eye health, MINGLE aims to adapt the successful aspects of WTL-Z [15] and the dietary intervention [16] to deliver it via an online program that facilitates social interaction among people with AMD. This will be achieved over two phases: 1) a small qualitative study (semi-structured interviews) with AMD patients to determine the barriers and facilitators to participating in such a program; and 2) applying the findings from phase 1 to inform the adaptation of the existing Walk and Talk for your Life program to develop the MINGLE program, followed by a pilot study to evaluate the feasibility and acceptability of MINGLE.

Project Design

**1) Participants and recruitment**

Participants will be recruited from a Sydney West Retina private eye clinic in Westmead. This eye clinic sees 800+ patients with AMD and ensures that recruitment targets will be met. Clinic staff will identify potentially eligible patients in the waiting room who have an AMD diagnosis and inform the research assistant accordingly. The research assistant will then approach the patients individually to discuss the study and assess additional eligibility criteria such as age and phase-specific criteria including administering the PAR-Q for phase 2. In the case of COVID-19 restrictions preventing face-to-face recruitment, clinic staff have agreed in attached Letter of Support to provide the researcher with the contact details of interested AMD participants to be contacted via telephone. Patient telephone numbers will be securely provided to the Research Assistant via a password protected excel file stored on the clinic’s Dropbox account. Access to the Dropbox account will require direct email invitation. The Research Assistant will approach eligible patients to discuss the phase-specific study and provide the relevant Participant Information Statement. Phase 1 and 2 participants will have the opportunity to read over the respective information thoroughly and ask any questions either in person or over-the-phone prior to providing verbal consent to participate in Phase 1 or signing the consent form to participate in Phase 2. The signed consent form can be collected in person by the research assistant or via a reply-paid envelope provided to potential participants.

***Sample Size***

*Phase 1:* Recruitment of participants until data saturation achieved. According to a previous qualitative study conducted in people with AMD, approximately 30 participants were required to achieve this. The estimated timeframe for recruitment is 3 months.

*Phase 2:* At least 52 participants are required to achieve a pre-post change in loneliness , assuming a 0.4 effect size according to Cohen’s d and 80% power (G*Power 3.1 software). The estimated timeframe for recruitment is 6 months.

***Inclusion criteria***

*Phase 1 (semi-structured interviews) eligibility:* 1) aged ≥ 50 years with a diagnosis of any form of AMD, 2) fluent English, 3) consent to participate in the study (including audio recording).

*Phase 2 (pilot study) eligibility:* 1) aged ≥ 50 years with a diagnosis of any form of AMD, 2) fluent English, 3) access to a smart phone, tablet or laptop with a front-facing camera; 4) technical ability to use Zoom or have someone to help; 4) clearance to safely participate in the physical activity components of the intervention (i.e. adequate responses to the researcher-administered Physical Activity Readiness Questionnaire (PARQ+)[17] or a copy of written clearance from their physician to engage in physical activity; 5) written informed consent to participate.

*Phase 2 exclusion criteria:* 1) unable to ambulate/walk for exercise; 2) serious illness limiting their ability to exercise or complete the trial; 3) contraindications to exercise (i.e. failure to fulfil the prerequisites of the PARQ+); 4) uncontrolled hypertension (≥160/>90 mmHg); 5) signs or symptoms of alcohol/substance abuse and; 6) unable to commit to attending ≥80% of the sessions.

**2) Data Collection**

*Phase 1:* Face-to-face audio-recorded semi-structured interviews will be conducted with participants. The audio recordings will be transcribed verbatim by a transcribing service.

*Phase 2:* Feasibility will be assessed by keeping track of all correspondence with each participant to allow for reporting on: the number sessions attended, completed sessions (‘dose’ of intervention received), and reasons for missed sessions. The session content will be tracked via checklists completed after each session allowing for reporting on the extent to which the intervention content is delivered per protocol and percentage of participants engaging/completing the activities. A short feedback form to determine the acceptability of the program will include open- and close-ended questions covering level of satisfaction with the program, self-determined adherence to the program, and general feedback (e.g. suggestions to improve the program). Participants will also complete a pre-post intervention online questionnaire. The online questionnaire will contain questions about demographics and socioeconomic status (SES), and validated scales will assess loneliness [18], depressive symptoms [19], physical activity level [20], dietary intake [21], quality of life, vision function [22] and falls efficacy [23,24]. Participants who withdraw from the study will not have any additional information collected, however information already collected with be retained to ensure that the results of the research project can be measured properly.

**3) Data Management**

*Recruitment:* In the case of COVID-19 restrictions preventing in-person recruitment, the telephone numbers of interested AMD patients will be securely provided to the Research Assistant via a password protected excel file stored on the clinic’s Dropbox account. Access to the Dropbox account will require direct email invitation. Any patient who chooses not to participate will have their details permanently deleted from the excel file. Patients who do choose to participate will be considered ‘participants’ and their data will be managed as described below according to the Phase. At conclusion of the recruitment stage, the Research Assistant will permanently delete the excel file and notify the clinic staff to delete access to their Dropbox account.

*Phase 1:* Audio recordings and transcriptions will be stored on University’s CloudStor+ for the duration of the study. Upon completion of the study, data will be held for a minimum retention period of 5 years.

*Phase 2:* Data will be entered into REDCap – a secure web application for building and managing online surveys and databases. Upon completion of the study, data will be exported to the University’s CloudStor+ and stored for a minimum retention period of 5 years. Hardcopy data will be securely stored in a locked storage cabinet on Level 3.514, Australian Hearing Hub, 16 University Avenue, Macquarie University NSW 2109with access limited to the research team. This data will also be stored for a minimum retention period of 5 years, after which, the data will be disposed of by secure disposal.

**4) Intervention program (MINGLE)**

Using Phase 1 findings, MINGLE will be adapted from the existing WTL program and telehealth nutrition intervention. A draft form of MINGLE will involve the following: Participants will be divided into subgroups based on session timings (morning/afternoon). The MINGLE program will be delivered via Zoom. The research assistant will contact participants at least 24 hours before their scheduled session with a Zoom link and any other relevant material e.g., activity material. The research assistant’s phone number will also be provided in case any participant has difficulty accessing Zoom. Sessions will run for 60-minutes once/week over ten-weeks. Sessions will be structured as follows: 10 minutes informal socialising; 30 minutes physical activity session; 15 minutes nutrition education. The physical activity session will not require any equipment other than a chair and will be facilitated by the research assistant (with an exercise-related qualification) and a research student who will be enrolled in a medical/allied health degree. Each physical activity session will include: 2-minute mindful breathing, 12-minute warm-up (e.g. stretching and bodyweight strengthening exercises), 7-minute moderate-to-vigorous intensity shadow boxing, and 9-minute cool down (e.g. bodyweight strengthening, balance exercises). Participants will be given a short break to use the facilities and drink water before commencing the 15-minute nutrition education session with a focus on evidence-based topics e.g. Age-Related Eye Disease Study (AREDS) supplementation, key food groups, and dietary patterns. The format of nutrition education will vary and range from presentations by the research assistant to interactive activity and/or discussion.

**5) Data Analysis**

*Phase 1:* Verbatim transcripts of the semi-structured interviews will be analysed iteratively to ensure analytic reflexivity. Transcripts will be coded separately by the research assistant and research student, using the Capability, Opportunity, Motivation and Behaviour (COM-B) model of the behaviour change wheel and discussed with the investigators. The research assistant and student will then establish themes under each subset of the COM-B model relating to barriers and facilitators to participating in an interactive online program. Phase 1 findings will inform the design of MINGLE.

*Phase 2:* Statistical analysis will be carried out using IBM SPSS Statistics V.25. Descriptive statistics will be used to assess the acceptability and feasibility of MINGLE. T-tests will be carried out to assess the pre-post intervention improvement in loneliness,quality of life, falls efficacy, physical activity, and dietary intakes.

Potential Risks

*1. Informed consent:* All participants recruited into this study will be given a Participant Information Statement that will include a clear explanation of the study objectives, risks of participation, and potential benefits of the study. Participants will be required to read the consent form and will be given opportunity to ask/clarify any questions before signing the consent form. All participation is voluntary, and participants will be able to withdraw from the study at any time.

*2. Confidentiality:* Data for all study participants will be strictly confidential. The information collected via semi-structured interviews and pre-post intervention surveys will be limited to an access-only database on REDCap - a secure platform for managing online databases. No access to identifiers will be permitted to any persons other than investigators and study staff. No subject identifiers will be released, and no reports will be published that could identify individual subjects.

*3. Data storage:* At the conclusion of the project, subject identifiers will be removed from study records. These will be coded with a subject number and ultimately destroyed. The database will be de-identified. Software identity links will be kept on a separate password-protected computer in a lockable office on the Macquarie University server.

*4. Risk of injury from exercise while participating in the intervention:* This risk will be minimised and managed by ensuring all participants show on camera that there is sufficient space to optimally perform the exercises. Participants will also be reminded in each session to make sure the tables and/or chairs they use to hold on to for balance are stable and secure prior to starting the exercise component. The research team member will also be trained and supervised by Dr Macniven and will: 1) demonstrate all exercises to the participants, 2) continually monitor all participants to ensure exercises are performed correctly to prevent injury. Modified exercises will also be available (e.g. low impact) for any participant with pre-existing injury/physical limitations.

*5. Verbal abuse:* Participants may offend one another during the intervention; therefore, participants and the research team will sign a Code of Conduct, outlining the rules of the study. This Code of Conduct will state how participants are to treat each other. Participants who violate this Code of Conduct will be removed from the study.

*6. Disclosure of sensitive personal information:* This risk will be minimised by ensuring all participants and research team members involved in the intervention sign the Code of Conduct which states “Do not disclose sensitive information about other participants involved in the study”. Participants who violate this Code will be removed from the study.

Outcome Measures

*Survey Outcomes*

The primary survey outcome is a significant change in loneliness according to the De Jong Gierveld Scale [18]. The secondary outcomes include significant changes in depression; quality of life; falls self-efficacy; physical activity level and overall diet using the AQoL-8D’s mental health dimension , overall AQoL-8D, FES-I [25], Active Australia Survey [26], and SDQ-AMD [21], respectively.

*Process Outcomes*

The key process outcome of this study is the development of an acceptable and feasible novel holistic online intervention with a focus on socialisation, physical activity, and nutrition education to specifically improve the wellbeing of people with AMD. The program is particularly attractive as online administration is more cost-effective than in-person engagement and offers convenience and COVID-19 safety to that participant as they can participate in the comfort of their own home. The study will also report on screening, eligibility, consent, retention, completion, and intervention adherence rates.

Results, Outcomes and Future Plans

A one page summary of the findings from this pilot study will be shared with study participants via post or email for who opt in to receive this on their consent form. Study findings will also be disseminated through publications in peer-reviewed journals and presentations at conferences. Pilot study findings will also be used to optimise the design and conduct of a larger randomised controlled trial. If the RCT shows efficacy and cost-effectiveness of the program, we will liaise with the MDFA to discuss the potential for a larger-scale roll-out to make the program publicly available.

References

[1] Mitchell P, Liew G, Gopinath B, Wong TY. Age-related macular degeneration. The Lancet 2018;392:1147–59. https://doi.org/10.1016/S0140-6736(18)31550-2.

[2] Dinu M, Pagliai G, Casini A, Sofi F. Food groups and risk of age-related macular degeneration: a systematic review with meta-analysis. European Journal of Nutrition 2019;58:2123–43. https://doi.org/10.1007/s00394-018-1771-5.

[3] Broadhead GK, Grigg JR, Chang AA, McCluskey P. Dietary modification and supplementation for the treatment of age-related macular degeneration. Nutrition Reviews 2015;73:448–62. https://doi.org/10.1093/nutrit/nuv005.

[4] McGuinness MB, Le J, Mitchell P, Gopinath B, Cerin E, Saksens NTM, et al. Physical Activity and Age-related Macular Degeneration: A Systematic Literature Review and Meta-analysis. American Journal of Ophthalmology 2017;180:29–38. https://doi.org/10.1016/j.ajo.2017.05.016.

[5] Casten RJ, Rovner BW. Update on depression and age-related macular degeneration. Current Opinion in Ophthalmology 2013;24:239–43. https://doi.org/10.1097/ICU.0b013e32835f8e55.

[6] Alma MA, van der Mei SF, Feitsma WN, Groothoff JW, van Tilburg TG, Suurmeijer TPBM. Loneliness and self-management abilities in the visually impaired elderly. Journal of Aging and Health 2011;23:843–61. https://doi.org/10.1177/0898264311399758.

[7] Nolen-Hoeksema S, Ahrens C. Age differences and similarities in the correlates of depressive symptoms. Psychology and Aging 2002;17:116–24. https://doi.org/10.1037//0882-7974.17.1.116.

[8] Schrempft S, Jackowska M, Hamer M, Steptoe A. Associations between social isolation, loneliness, and objective physical activity in older men and women. BMC Public Health 2019;19:74. https://doi.org/10.1186/s12889-019-6424-y.

[9] Sutin AR, Stephan Y, Luchetti M, Terracciano A. Loneliness and Risk of Dementia. The Journals of Gerontology Series B, Psychological Sciences and Social Sciences 2020;75:1414–22. https://doi.org/10.1093/geronb/gby112.

[10] Hawkley LC, Cacioppo JT. Loneliness matters: a theoretical and empirical review of consequences and  mechanisms. Annals of Behavioral Medicine : A Publication of the Society of Behavioral Medicine 2010;40:218–27. https://doi.org/10.1007/s12160-010-9210-8.

[11] Cacioppo JT, Hawkley LC, Crawford LE, Ernst JM, Burleson MH, Kowalewski RB, et al. Loneliness and Health: Potential Mechanisms. Psychosomatic Medicine 2002;64.

[12] Fiske A, Wetherell JL, Gatz M. Depression in older adults. Annual Review of Clinical Psychology 2009;5:363–89. https://doi.org/10.1146/annurev.clinpsy.032408.153621.

[13] Cattan M, White M, Bond J, Learmouth A. Preventing social isolation and loneliness among older people: a systematic review of health promotion interventions 2005.

[14] Hwang J, Wang L, Jones C. Tackling social isolation and loneliness through community exercise programs for seniors COMMENTARY. vol. 8. 2016.

[15] Bender N, Elliot A, Jones CA. Walk and Talk for Your Life hosted over Zoom: A pilot study on the effects of an online, videoconference-based group exercise and health discussion intervention on loneliness in older adults. Unpublished Manuscript 2020.

[16] Tang D, Mitchell P, Liew G, Burlutsky G, Flood VM, Gopinath B. Telephone-Delivered Dietary Intervention in Patients with Age-Related Macular Degeneration: 3-Month Post-Intervention Findings of a Randomised Controlled Trial. Nutrients 2020;12:3083. https://doi.org/10.3390/nu12103083.

[17] Bredin SSD, Gledhill N, Jamnik VK, Warburton DER. PAR-Q+ and ePARmed-X+: new risk stratification and physical activity clearance strategy for physicians and patients alike. Canadian Family Physician Medecin de Famille Canadien 2013;59:273–7.

[18] Gierveld JDJ, Tilburg T van. A 6-Item Scale for Overall, Emotional, and Social Loneliness. Research on Aging 2006;28:582–98. https://doi.org/10.1177/0164027506289723.

[19] Ishihara T, Terada S. [Geriatric Depression Scale (GDS)]. Nihon Rinsho Japanese Journal of Clinical Medicine 2011;69 Suppl 8:455–8. https://doi.org/10.1300/j018v05n01_09.

[20] Merom D, Delbaere K, Cumming R, Voukelatos A, Rissel C, van der Ploeg HP, et al. Incidental and Planned Exercise Questionnaire for seniors: validity and  responsiveness. Medicine and Science in Sports and Exercise 2014;46:947–54. https://doi.org/10.1249/MSS.0000000000000196.

[21] Tang D, Mitchell P, Liew G, Burlutsky G, Flood V, Gopinath B. Evaluation of a Novel Tool for Screening Inadequate Food Intake in Age-Related Macular Degeneration Patients. Nutrients 2019;11:3031. https://doi.org/10.3390/nu11123031.

[22] Mangione CM, Lee PP, Gutierrez PR, Spritzer K, Berry S, Hays RD. Development of the 25-item National Eye Institute Visual Function Questionnaire. Archives of Ophthalmology 2001;119:1050–8. https://doi.org/10.1001/archopht.119.7.1050.

[23] Promotion Unit H, Health Branch P. SUPPLEMENTARY DATA Translators’/Interviewers’ notes for FES-I. n.d.

[24] Promotion Unit H, Health Branch P. Questionnaire to assess attitudes to balance and falling-related interventions Why measure attitudes to interventions? n.d.

[25] Delbaere K, Close JCT, Mikolaizak AS, Sachdev PS, Brodaty H, Lord SR. The falls efficacy scale international (FES-I). A comprehensive longitudinal validation study. Age and Ageing 2010;39:210–6. https://doi.org/10.1093/ageing/afp225.

[26] The Active Australia Survey: a guide and manual for implementation, analysis and reporting, Summary - Australian Institute of Health and Welfare n.d. https://www.aihw.gov.au/reports/physical-activity/active-australia-survey/summary (accessed June 28, 2021).
