# Supplementary material for: Development, implementation and evaluation of the online Movement, Interaction and Nutrition for Greater Lifestyles in the Elderly (MINGLE) program: The protocol for a pilot trial
Source: PLoS One. 2022 May 12;17(5):e0267581. doi: 10.1371/journal.pone.0267581 (PMC9097998; doi:10.1371/journal.pone.0267581)
Supplement: Attachment — Submitted filename: Response to reviewers.docx [file pone.0267581.s003.docx]

Response to Reviewers

**Reviewer #1**

**Comment 1. The project is well thought of and can provide significant support for people with AMD. However, I have major and minor concerns about the protocol. First, the Qualitative component of the project as a sequential exploratory design might not provide clear guidance to the quantitative part. The project is looking for specific answers regarding MINGLE program (duration, frequency..etc.); however, participants do not know anything about it. Therefore, it is unlikely that their input will help in structuring phase 2.**

Response 1. Thank you for taking the time to review our manuscript. We acknowledge that the qualitative component of the project may not provide clear guidance to quantitative component, however we feel it is still important to engage with participants about the program to identify potential barriers and facilitators to participating in MINGLE. Responses to specific questions like duration and frequency may not reflect the general population of older adults with AMD but can be taken into consideration to improve the acceptability of the program as participants of the qualitative component will also be invited to participate in the quantitative component.

**Comment 2. Additionally, the analysis section of Phase1 needs further information about who will conduct the interviews (background and qualification), where the interview will be held, who will do the thematic analysis, and whether it is inductive or deductive. Moreover, it is advisable to provide a topic guide for the semi-structured interview.**

Response 2. Thank you for your comment. We have taken on board your suggestions and have elaborated on the analysis section of Phase 1 as below. A topic guide has also been included in Table 1.

Page 9 “Interviews will be conducted in-person in a private room at the eye clinic or over the telephone depending on the participant’s preference and/or COVID-19 restrictions.”

Page 16 “Two members of the research team with a vision science and/or medical background will deductively code the transcripts using content analysis (Vaismoradi et al., 2013)…”

**Comment 3. There is no information on the minimal visual requirement in the inclusion criteria since you use a video conferencing platform (Zoom).**

Response 3. Thank you for your suggestion to include a visual requirement in the inclusion criteria for Phase 2. We agree that this is a valid criterion to reduce the risk of injury while following the exercises being demonstrated on Zoom. Therefore, we have no included that people with best-corrected visual acuity of 6/60 (legal blindness) or less in both eyes will be excluded from the study.

**Comment 4. The aim of the project was (feasibility, acceptability, and efficacy). While the "Attitude to Falls-Related Intervention Scale" could provide quantitative information about the "acceptability," I am not sure it could guide feasibility. Can you state specific parameters that can determine the feasibility for a larger study; like attrition rate, follow-up and qualitative data regarding length of the study, outcome measure appropriateness (e.g., length).**

Response 4. Thank you for your comment. We have taken your suggestions on board and have included specific parameters that can determine the feasibility for a larger study.

Page 15 “Data collected on the abovementioned measures will be used to evaluate the program’s preliminary efficacy and its acceptability and feasibility. Specifically, this includes an evaluation of: 1) recruitment strategies – identifying the reach of each recruitment source; 2) recruitment rates at each stage of the project i.e., total number who expressed an interest in Phase 1 or 2, proportion who were eligible, provided consent, and completed the baseline questionnaire; 3) program adherence based on self-reported adherence and the Research Assistant’s evaluation during each session; 4) attrition rate; 5) pre-post responses to the AFRIS questionnaire; and 6) qualitative data obtained from the feedback survey and focus group discussion immediately post-intervention (Table 1). The feedback survey includes Likert scales to rate overall satisfaction with the program and adherence to the nutritional advice provided, two Yes/No questions to determine whether participants would recommend the program to a friend and if the program was worthwhile, and two open-ended questions to identify the most useful or valuable components of the program, and for additional suggestions to improve the program…”

**Comment 5. Use APA style to cite "ZOOM"**

Response 5. Thank you for your comment. We have now provided APA style citation for Zoom as “Zoom Communications Inc. (2016)”.

**Comment 6. Bender et al. should not be cited in the main text. use APA style to cite it in the reference section.**

Response 6. Thank you for your comment. After reviewing formatting feedback from the Editor, we have left the citation in-text as unpublished work cannot be cited in the reference list for this journal.

**Reviewer #2**

**Comment 1. The physical activity component needs more details as what type of exercises will be done and how would you determine the difficulty of the exercises!**

Response 1. Thank you for taking the time to review our manuscript. We have taken on board your advice and have provided more detail on the type of exercises included and how the intensity of the exercises will be assessed.

Page 11 “Each physical activity session will include: 2-minute mindful breathing, 12-minute warm-up (e.g., stretching and bodyweight strengthening exercises), 7-minute moderate-to-vigorous intensity shadow boxing, and 9-minute cool down (e.g., bodyweight strengthening, balance exercises). The intensity of the exercises will be self-evaluated using the Borg Rating of Perceived Exertion (RPE) Scale (Williams, 2017).”
